# Evolution, Current Trends, and Latest Advances of Endoscopic Spine Surgery

**DOI:** 10.3390/jcm13113208

**Published:** 2024-05-29

**Authors:** Sharvari Gunjotikar, Malcolm Pestonji, Masato Tanaka, Tadashi Komatsubara, Shashank J. Ekade, Ahmed Majid Heydar, Huynh Kim Hieu

**Affiliations:** 1 Department of Orthopedic Surgery, Okayama Rosai Hospital, 1-10-25 Chikkomidorimachi, Minami Ward, Okayama 702-8055, Japan; sharvarigunjotikar@gmail.com (S.G.); t.komatsubara1982@gmail.com (T.K.); drshashankjekade@gmail.com (S.J.E.); dr.a.heydar@gmail.com (A.M.H.); hkhieu@ctump.edu.vn (H.K.H.); 2 Department of Orthopedic Surgery, Golden Park Hospital and Endoscopic Spine Foundation India, Vasai West, Thane 401202, Maharashtra, India; malcolmpestonji64@gmail.com

**Keywords:** endoscopic spinal surgery, full endoscopy, biportal endoscopy, lumbar endoscopy, thoracic endoscopy, cervical endoscopy

## Abstract

**Background**: The aging of the population in developing and developed countries has led to a significant increase in the health burden of spinal diseases. These elderly patients often have a number of medical comorbidities due to aging. The need for minimally invasive techniques to address spinal disorders in this elderly population group cannot be stressed enough. Minimally invasive spine surgery (MISS) has several proven benefits, such as minimal muscle trauma, minimal bony resection, lesser postoperative pain, decreased infection rate, and shorter hospital stay. **Methods**: A comprehensive search of the literature was performed using PubMed. **Results**: Over the past 40 years, constant efforts have been made to develop newer techniques of spine surgery. Endoscopic spine surgery is one such subset of MISS, which has all the benefits of modern MISS. Endoscopic spine surgery was initially limited only to the treatment of lumbar disc herniation. With improvements in optics, endoscopes, endoscopic drills and shavers, and irrigation pumps, there has been a paradigm shift. Endoscopic spine surgery can now be performed with high magnification, thus allowing its application not only to lumbar spinal stenosis but also to spinal fusion surgeries and cervical and thoracic pathology as well. There has been increasing evidence in support of these newer techniques of spine surgery. **Conclusions**: For this report, we studied the currently available literature and outlined the historical evolution of endoscopic spine surgery, the various endoscopic systems and techniques available, and the current applications of endoscopic techniques as an alternative to traditional spinal surgery.

## 1. Introduction

With advancements in medical care and increased life expectancy of the aging population, the global burden of spinal disease has increased [1]. Spine-related disorders significantly affect the quality of life (QOL) and ability to perform daily living activities among the elderly [2]. Elderly patients with spinal disorders often suffer from numerous comorbidities and medical problems, further complicating surgical treatment and functional outcomes [1,3,4]. In an attempt to decrease the morbidity associated with conventional open spine surgery, numerous advances have been made in the field of minimally invasive spine (MIS) surgery. MIS surgery has several advantages, such as minimal soft tissue trauma, lesser blood loss, decreased infection rates, earlier rehabilitation, shorter hospital stays, and better functional outcomes [5]. Despite these advantages, the long learning curve, the need for special instruments and types of equipment, high costs, lack of tactile sensation and biplanar imaging, some complications that are hard to treat, and more radiation to the surgeon and surgical team are the disadvantages of MIS surgery [6].

Endoscopic spine surgery is an evolving subset of MIS surgery with ever-growing indications [7]. Over the last four decades, there has been tremendous development in the field of endoscopic spine surgery. Endoscopic techniques have evolved from the earlier attempts of percutaneous nucleotomy to modern techniques of full endoscopy and biportal endoscopic decompression. With the development of specialized instrumentation and high-resolution imaging, endoscopic spine surgery, initially limited to lumbar discectomies, can now be used to treat a wide range of spinal pathologies such as spinal stenosis, instability, thoracic and cervical myelopathy, infections, intradural tumors, etc. [8,9,10].

With this report, we use PubMed to search the relevant important reports and aim to outline the historical evolution of endoscopic techniques for spine surgery, the present applications of endoscopic spine surgery in clinical practice, and the latest advances in this field.

## 2. Evolution of Endoscopic Techniques for Spine Surgery (Table 1)

The earliest account of percutaneous decompression techniques dates back to Kambin in 1973 and Hijikata in 1975, who described their technique of percutaneous nucleotomy, which was an indirect non-visualized decompression through the postero-lateral approach using fluoroscopy. Kambin used a Craig cannula (5.5 mm), and Hijikata used a 2.6 mm cannula, respectively [11,12]. The next advancement to percutaneous nucleotomy was the addition of endoscopes, and in 1983, Kambin described his technique of percutaneous arthroscopic discectomy [13]. Subsequently, in 1990, he described a triangular safe zone bordered by the exiting root anteriorly, the traversing root medially, and the superior endplate of the lower lumbar vertebra inferiorly [14]. The description of this radiographic safe working zone allowed the introduction of larger instruments and working channels in closer proximity to the foraminal pathology without injuring the nerve root and thus led to further advancements in the field of endoscopic spine surgery.

In 1997, Foley described the technique of micro-endoscopic discectomy, which is one of the most popular techniques in discectomy. He used a 25-degree scope through a 16 mm tubular retractor to achieve decompression for far lateral disc herniation [15,16]. In the same year, Destandau’s Endospine technique was introduced by Dr J. Destandau, based on the principle of laparoscopic triangulation between an endoscope and suction with a working instrument. The system is composed of three tubes: one for the endoscope, one for aspiration, and the largest one for standard surgical instruments [17,18]. In the 1990s, Yeung developed an operating spine scope with a working channel and introduced beveled and slotted cannulas and, subsequently, allowed for direct visualization and surgical removal of disc material and foraminal decompression (foraminoplasty) through a single port [19,20]. They called their technique the “inside-out technique” of endoscopic spine surgery, where the working cannula was placed inside the intervertebral disc [21]. Subsequently, in an attempt to avoid irritation of the nerve root in cases of foraminal stenosis, Thomas Hoogland described the “outside-in technique”, where the working cannula was placed in the neural foramen after widening it using reamers [22].

Even though transforaminal endoscopic techniques were popular, there were several technical challenges for transforaminal access at the L5-S1 level owing to anatomical constraints such as high iliac crest, large L5 transverse process, large facet, narrow disc space, and neural foramen [23,24]. In an attempt to overcome these technical difficulties, Choi et al. described the technique of percutaneous endoscopic interlaminar discectomy using a rigid working-channel endoscope [25]. Irrespective of the technique used, the above endoscopic spine surgeries are performed through a single incision involving a light source, irrigation, and instrumentation. Despite the use of superior imaging, visualization is restricted, and technical difficulties may be encountered by surgeons, which are of relevance in severely stenotic canals or in cases requiring bilateral decompression [26]. Unilateral biportal endoscopic spinal surgery (UBE) or percutaneous biportal endoscopic decompression (PBED) is the combination of integrated open and endoscopic spinal surgery, which can lessen the impact of the limitations [27]. Unlike other endoscopic techniques, this technique utilizes 2 independent portals, one for the introduction of the endoscope and the other for the introduction of surgical instruments.

**Table 1:** Historical evolution of endoscopic technique.

Year	Author	Technique Described
1973	Kambin [11]	Percutaneous nucleotomy with Craig cannula (5 mm) Fluoroscopy guided without visualization
1975	Hijikata [12]	Percutaneous nucleotomy (2.6 mm cannula) Fluoroscopy guided without visualization
1983	Kambin [13]	Percutaneous arthroscopic discectomy
1997	Foley [15]	Micro-endoscopic discectomy
1999	Yeung [19]	YESS—inside-out technique
1999	Destandau [17]	Destandau’s Endospine Technique
2005	Hoogland [22]	Transforaminal Endoscopy—Outside-in technique
2006	Choi [25]	Interlaminar approach for L5-S1 level
2016	Eum et al. [27]	Unilateral Biportal Endoscopy (UBE)/Percutaneous Biportal Endoscopic Decompression (PBED)

## 3. Lumbar Spine Endoscopy

### 3.1. Micro-Endoscopic Discectomy and Decompression (Table 2)

**Figure 1:**
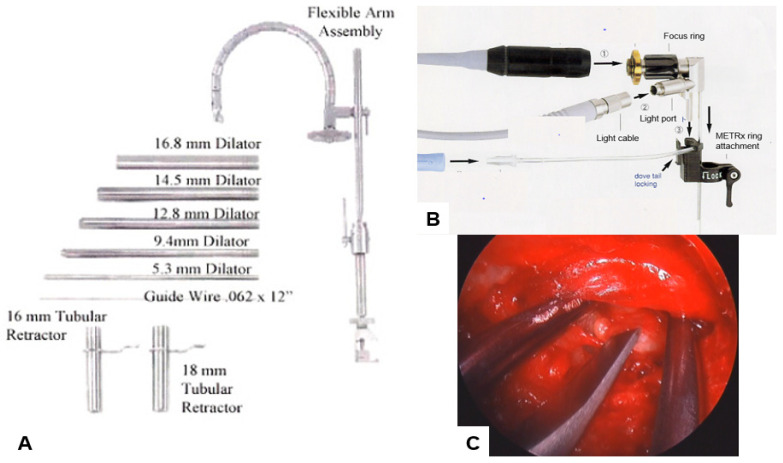
MED, (**A**) METRx system (**B**) Scope and attachment, (**C**) Endoscopy image.

Micro-endoscopic discectomy is a minimally invasive surgery technique that was initially described in 1997 [15]. It allows surgeons to work with two hands through a small-diameter, operating table–mounted tubular retractor and a 25-degree endoscope [15,16] (Figure 1). Compared to conventional open discectomy, micro-endoscopic discectomy (MED) has several advantages, such as less blood loss, cosmesis, shorter hospital stays, and early return to work [16]. A study of 150 consecutive patients treated with MED in 2005 confirmed these findings [28]. MED technique has been used not only for paracentral disc herniation but also for all types, including far lateral, cephalad, caudal migrated, and recurrent disc herniation [29,30].

Due to the advantages of MED, in 2002, Khoo and Fessler modified the MED technique and used it to treat 25 patients with LSS [31]. Compared to the open technique, the micro-endoscopic decompression group had a statistical decrease in operative blood loss, postoperative narcotic requirement, and length of hospital stay (42 h versus 94 h) [31]. Even today, Micro-endoscopic discectomy and decompression are some of the most commonly performed techniques for the treatment of lumbar disc herniations and lumbar spinal stenosis.

**Table 2:** Surgical results of micro-endoscopic discectomy and decompression.

Author	Sample	Approach	Follow-Up	Outcomes	Complications
Perez-Cruet 2002 [28]	150	Paramedian	12 months	Mean operative time (min) 97 Mean hospital stay (hours) 7.7 (range, 2–24) Mean time to return to work (days) 17	Dural tears 8/150 (5%) recurrence 4/150 Pseudomeningocoele 1/150 (0.7%) Surgical site infection 1/150 (0.7%)
Khoo 2002 [31]	25 vs. 25 open	Paramedian	12 months	Lesser blood loss 44 mL for med vs. 193 mL Shorter hospital stay 42 h vs. 94 h	Additional fusion surgery 0% vs. 12% Transfusion 0% vs. 8% Dural tear 16% vs. 8%
Ikuta 2005 [32]	47 vs. 29 Micro discectomy	Paramedian	22 months	Rate of recovery 72% (improvement in JOA score) (38/47)	Higher complication rate compared to microdiscectomy
Wu 2006 [33]	873 vs. 358 open	Not specified	28 months vs. 31 months for open	Earlier return to work Shorter hospital stay Shorter operation time Lesser blood loss Lesser analgesic need	35/873 for MED vs. 19/358
Fukushi 2015 [34]	58 vs. 39 open	Midline	42 months	Similar improvement in JOA and similar patient satisfaction	Higher rate of infection.
Wu 2020 [35]	82 vs. 52 Full endoscopy	Paramedian	20 months	Similar improvement in VAS for leg pain Higher VAS for low back pain and ODI	Dysesthesia 0% vs. 1.9%, Dural tear 2.4%vs. 1.9%, Urinary retention 1.2% vs. 0%, Total 3.85% vs. 3.66%
Iwai 2020 [36]	60 vs. 54 Micro-endoscopic	Paramedian	3 months	Shorter operating time but longer hospital stay compared to biportal endoscopy. Similar VAS/NRS in both groups	Dural tear 5.6% vs. 1.8% Hematoma 3.3% vs. 13.0%
Ito 2021 [37]	139 vs. 42 Biportal Endoscopy	Paramedian	6 months	Similar improvement in VAS for low back pain and leg pain, OD	Dural tear 5.8% vs. 4.7%, Hematoma 3.6% vs. 0%, Re-operation 1.4% vs. 0%

### 3.2. Destandau’s Endospine Technique

**Figure 2:**
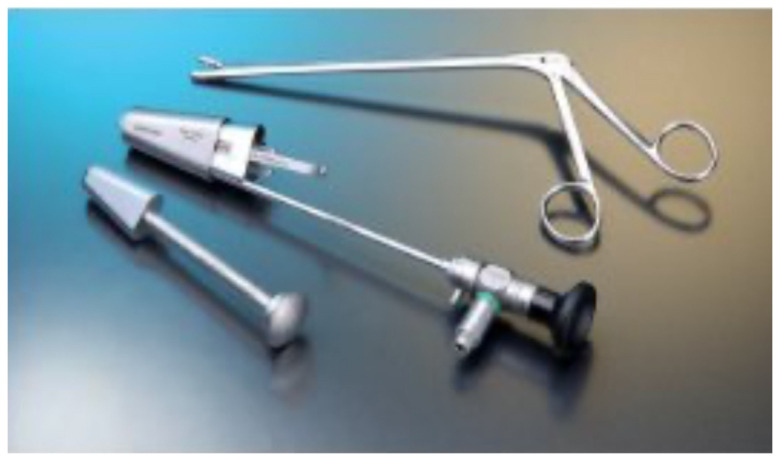
Instruments of Destandau’s endospine technique.

This technique requires the use of a specialized system called the Destandau Endospine System (Karl Storz, Tuttlingen, Germany, Figure 2), which comprises an endospine tube, trocar, and a working insert. The working insert comprises four ports—4-mm endoscope (0°), 4-mm suction cannula, 8-mm for the working instrument, and nerve root retractor [38]. There are numerous applications of the Destandau system, ranging from degenerative spinal conditions to intradural tumor surgery [22].

### 3.3. Transforaminal Endoscopy

**Figure 3:**
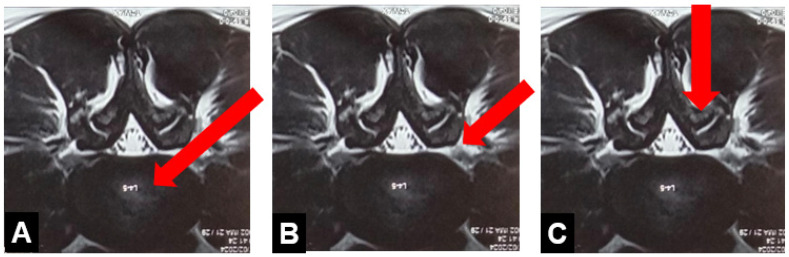
Three kinds of endoscopic approaches. (**A**) Inside-out approach—Starts from the disc to the epidural space, (**B**) Outside-in approach—Starts from the epidural space, with or without foraminoplasty, (**C**) Interlaminar approach. Red arrows indicate scope positions.

The transforaminal approach was the first approach that was used by the pioneers of full endoscopic spine surgery techniques [8]. The key factor for transforaminal decompression is the safe docking of the endoscope, which requires careful analysis of preoperative radiologic imaging to evaluate the angle of approach required to retrieve fragments and understand the anatomic constraints in the transforaminal approach [8]. Two popular techniques are the “inside-out technique” [21] by Yeung and the “outside-in technique” described by Hoogland [22] (Figure 3). Although it was initially believed that the transforaminal approach has several limitations, the literature suggests that this technique can be applied successfully to treat migrated disc herniations and foraminal pathology such as foramina disc herniations and foraminal stenosis [39,40]. Literature suggests that challenging cases like highly migrated disc herniations can be operated using the transformational endoscopic lumbar discectomy (TELD) by performing partial resection of the pedicle and enlargement of the foramen [41]. TELF can be used to treat foraminal stenosis while maintaining favorable long-term outcomes without the need for fusion in the vast majority of patients [42]. Several cadaveric studies also showed confirmed feasibility and efficacy of percutaneous endoscopic lumbar foraminoplasty/TELF [43,44].

When used for revision spine surgery, TELF and TELD are associated with minimal blood loss, lesser scar tissue formation, and similar operating time compared to primary spine surgeries [45,46]. In addition to other advantages of this technique, such as shorter operation time, minimal blood loss, and less muscle trauma, there are several published reports of transforaminal endoscopic spine surgery performed in awake patients under local anesthesia [47,48]. Therefore, transforaminal endoscopic spine surgery is a viable surgical treatment option for patients with severe medical comorbidities who are medically unfit to undergo surgery under general anesthesia.

### 3.4. Interlaminar Endoscopy

#### 3.4.1. Interlaminar Endoscopic Lumbar Discectomy (IELD)

Due to the technical difficulty of using the transforaminal approach at the L5-S1 level due to anatomical constraints, the interlaminar approach was advocated [25]. Due to its similar orientation to conventional posterior decompression techniques, the interlaminar approach, though initially described for the L5-S1 level, was popularized for surgical treatment of lumbar canal stenosis (LCS) even at higher lumbar levels.

The patients undergoing IELD have significant advantages such as lower immediate postoperative back pain, shorter operation time, and rapid return to work [49]. Reuten et al. followed up 178 patients who underwent either interlaminar endoscopic lumbar discectomy (IELD) or microsurgical discectomy and concluded that full endoscopic surgery is a sufficient and safe alternative to microsurgical procedures with minimal soft tissue trauma [50].

The interlaminar approach can also be used to treat lumbar spinal stenosis. Lumbar endoscopic—unilateral laminotomy and bilateral decompression (LE-ULBD), when compared to microscopic ULBD, showed comparable clinical and radiological outcomes. The endoscopic approach might further minimize tissue injury and enhance postoperative recovery [51,52]. McGrath et al., in a similar study, found that lumbar endoscopic unilateral laminotomy for bilateral decompression is a safe and effective surgical procedure with a favorable complication profile and patient outcomes [53].

#### 3.4.2. Percutaneous Biportal Endoscopic Discectomy and Decompression (PBED)

**Figure 4:**
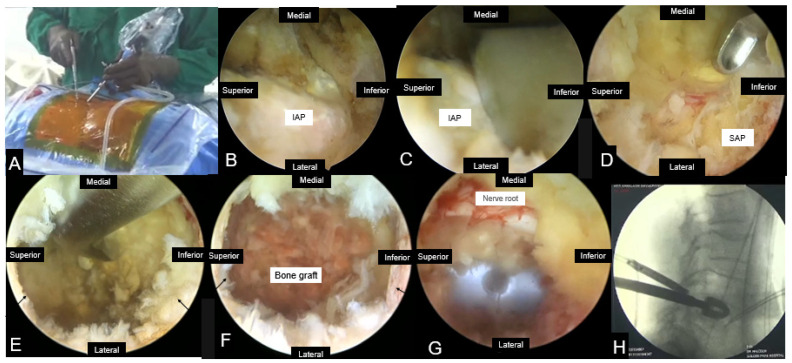
Unilateral biportal endoscopic transforaminal lumbar interbody fusion. (**A**) Scopic and Instrument portals, (**B**) Endoscopic view after soft tissue dissection, (**C**) Osteotomy of inferior articular process (IAP), (**D**) Resection of the tip of superior articular process (SAP), (**E**,**F**) Removal of disc and preparation of disc space, (**G**) Endoscopic view showing insertion of cage, (**H**) Final position of cage. This step is followed by insertion of percutaneous pedicle screws under fluoroscopic guidance.

The PBED technique involves using one portal for the endoscope and the other for the introduction of instruments for performing decompression (Figure 4). In recent years, this technique of spinal surgery has gained tremendous popularity due to the advantages of familiarity of surgeons with a posterior approach, free dexterity, and the use of conventional instruments used for open spine surgery [54,55,56]. It is generally believed that UBE surgery has the advantages of a wider field of vision, minimal muscle damage, and faster recovery [57]. The learning curve of this technique is relatively shorter than that of uniportal full endoscopic surgery, which is another advantage of the UBE technique [58].

UBE discectomy is a good treatment choice for lumbar disc herniation [59]. The biportal endoscopic technique can also be used to treat foraminal pathologies, such as foraminal stenosis and far lateral disc herniation, using the extraforaminal technique described by Ann [55].

A number of studies have compared the intra-operative and postoperative outcomes following PBED. Compared to ILED, PBED has superior results, such as shorter operation time, better central canal decompression, and less violation of the facet joints [60]. PBED has also demonstrated favorable clinical outcomes for revision surgery, such as lower postoperative pain, and has outcomes comparable to primary PBED, thus suggesting the advantage of PBED for revision spine surgeries [26,61] (Table 3).

#### 3.4.3. Endoscopic Lumbar Interbody Fusion

Current literature in endoscopic fusion can be broadly divided into transforaminal lumbar interbody fusion by the Kambin triangle ventral to the facet joint with or without foraminoplasty, transforaminal lumbar interbody fusion through posterolateral approach by uniportal or biportal endoscopy, through the resected facet joint and endoscopic-assisted lateral lumbar interbody fusion [8]. The technique of transforaminal lumbar interbody fusion via Kambin’s triangle can be used as an alternative technique for spinal fusion in patients with severe medical comorbidities as it can be done under local anesthesia [68]. The technique is based on the principle of indirect decompression and has several advantages, such as short operative time, minimal blood loss, and the ability to perform visualized preparation of the end plates. However, the disadvantages are the possibility of injuring the exiting nerve root and radiation exposure [69].

Full endoscopic lumbar interbody fusion (FELIF) or percutaneous endoscopic lumbar interbody fusion is one of the most commonly performed endoscopic techniques of spinal fusion. This technique is usually characterized by the following: use of a working-channel endoscope containing the optical system and the working channel within the same thin tubular device; complete percutaneous access with a stab incision; and monoportal approach with constant saline irrigation [70].

It has the advantage that it allows direct decompression of pathology with a minimally invasive technique [69]. FE-PLIF via the interlaminar approach is known to have outcomes compared to other popular minimally invasive techniques like MIS-TLIF with minimal surgical trauma [71]. Endoscopic lumbar interbody fusion using percutaneous unilateral biportal endoscopic technique can achieve direct neural decompression similar to conventional open surgery and can be an alternative to minimally invasive LIF surgery for treating degenerative lumbar disease. However, long-term follow-up and larger clinical studies are needed to validate the clinical and radiological results of this surgery [54].

According to a recent meta-analysis, UBE-TLIF was superior to MIS-TLIF in terms of intra-operative blood loss, duration of hospital stay, VAS score for low back pain, and ODI score, but the operative time was longer than MIS-TLIF group. There were no significant differences between the two groups in terms of total complication rate, modified Macnab grading criteria, fusion rate, VAS score of leg pain, lumbar lordosis, and intervertebral disk height [72].

## 4. Thoracic Spine Endoscopy

Initially limited to the lumbar spine, in recent years, there has been a steady and significant interest in the application of endoscopy to cervical and thoracic spine pathologies. With better instrumentation and technology, spinal endoscopy is now a viable alternative to traditional open surgery that avoids the risks and complications associated with open thoracic spine surgery. The sizes and the number of working channels are the parameters typically used to categorize the different types of spinal endoscopy and much of the capabilities of these different systems and their inherent advantages and disadvantages are predicated upon these two factors [73]. The techniques most commonly employed are full endoscopy (transforaminal and posterior percutaneous), micro-endoscopy, and biportal endoscopy.

### 4.1. Full Endoscopy

The following full endoscopic classifications are currently recommended by the AO Spine group [74]. (1) Transforaminal endoscopic thoracic discectomy (TETD), (2) Thoracic endoscopic unilateral laminotomy for bilateral decompression (TE-ULBD), (3) Transpedicular endoscopic surgery. The most commonly employed approaches are transforaminal and interlaminar. Most reports describe a transforaminal approach for disc prolapse with interlaminar access used for the treatment of significant canal stenosis [75]. The results of endoscopic discectomy in the thoracic spine have been satisfactory, suggesting the possibility of a large-scale role of minimally invasive endoscopic techniques in the surgical management of thoracic disc herniations, including soft as well as calcified discs [76,77].

The use of endoscopy for other spinal diseases, such as infection and tumor, has been reported in several publications [78]. Yang et al., in a relatively large series of patients with tuberculosis, described the use of percutaneous decompression and fusion with allograft followed by percutaneous pedicle screw fixation and reported excellent outcomes, with 96% of patients achieving acceptable fusion [79]. The increasing number of publications in the last three years suggests that full endoscopy is the technique of choice in many centers and will become the universal standard of patient care [75].

### 4.2. Micro-Endoscopy

The thoracic micro-endoscopic discectomy (TMED) technique is a modification of the lumbar micro-endoscopic technique that has been used with success to treat stenosis as well as disc herniation [80]. This method has been implemented successfully to treat lateralized and central soft thoracic disc herniation causing radicular and myelopathic symptoms [81].

The TMED technique has several advantages over other traditional techniques for thoracic discectomy and include the following: avoidance of entering the thoracic cavity, minimal osseous and ligamentous removal, maintenance of disc integrity, avoidance of the need for thoracic fusion, and avoidance of extensive posterior muscle dissection [81]. TMED is a safe and effective treatment for surgical removal of herniated thoracic intervertebral discs and allows for a posterolateral approach to thoracic disc herniation without entry into the chest cavity that consistently gives access to most of the canal while requiring only a minimal amount of bone removal [80,82].

### 4.3. Biportal Endoscopy (UBE)

Thoracic laminectomy has traditionally been considered the gold standard for the treatment of thoracic OLF and stenosis. Post-surgery backache, paraspinal muscular atrophy, and instability are known complications of traditional open surgery, often requiring revision and fusion. The clinical outcomes of a conventional technique for thoracic OLF or thoracic spinal stenosis are frequently unsatisfactory [83].

To address these issues, unilateral biportal endoscopy (UBE) techniques for thoracic laminectomy have been developed and published, demonstrating various advantages over conventional thoracic laminectomy and reporting competent clinical results [84,85]. The main advantages of this approach are the independence of scope and instrument control as well as a greater degree of freedom for the positioning of the instruments [73]. Although UBE has grown in popularity in recent years, thoracic ULBD via UBE is technically difficult [86].

UBE decompression is a viable treatment alternative that can achieve satisfactory clinical results in patients with thoracic OLF [85,87]. While treating thoracic OLF or thoracic spinal stenosis, the UBE decompression technique with a unilateral approach and bilateral decompression appears to be safe and effective [86]. Although thoracic ULBD by UBE is not currently the standard treatment for thoracic OLF or thoracic spinal stenosis, this technique has the potential to be more widely used in the future.

## 5. Cervical Spine Endoscopy

The anterior percutaneous cervical discectomy was the prototype of cervical endoscopic surgery [88]. Subsequently, many variations and techniques have been developed to deal with cervical spine pathologies. Anterior cervical discectomy and fusion (ACDF) has long been considered the gold standard for cervical disc disease [89].

### 5.1. Posterior Endoscopic Cervical Foraminotomy and Laminectomy (Figure 5)

Posterior full endoscopic cervical foraminotomy and additional discectomy showed similar clinical outcomes to conventional ACDF [90]. Cervical motion was preserved better in posterior full endoscopic cervical foraminotomy and discectomy [91]. Patients who underwent endoscopic surgery had less blood loss, shorter operation times, and shorter hospital stays than those treated with conventional open foraminotomy [92]. Cervical myelopathy, which was earlier thought to be a contra-indication for endoscopic surgery, is now routinely treated with UBE and large-diameter full endoscopes.

Some studies have demonstrated that UBE laminectomy may be considered an excellent surgical alternative to treat cervical stenosis without the development of iatrogenic kyphosis [93]. It represents an effective method with excellent neurological and radiological outcomes with less soft tissue invasion, which translates into dramatically less postoperative axial pain and maintains postoperative cervical lordosis [93]. Wang et al. concluded in their study that UBE and Percutaneous posterior endoscopic discectomy were both safe and effective in the treatment of cervical spondylotic radiculopathy and were characterized by minimal trauma, no adverse impact on cervical stability, and few complications [94]. It is safe to say that cervical endoscopic surgeries have evolved and are no longer limited to dealing with just single-level disc disease.

### 5.2. Endoscopic Anterior Cervical Discectomy and Fusion (Figure 6)

Ahn et al. reported 5-year follow-up outcomes of anterior full endoscopic discectomy for soft disc herniation and showed comparable results with conventional ACDF [95]. Recently, endoscopic ACDF has also been performed, but no concrete data are currently available to prove its efficacy (Table 4).

## 6. Craniovertebral Junction (CVJ) Endoscopy

**Figure 7:**
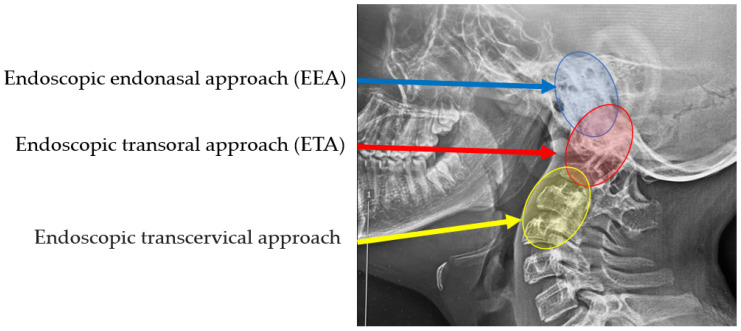
Three different endoscopic approaches to craniovertebral junction.

The craniovertebral junction (CVJ) is an important structure because this part includes the medulla and multiple cranial nerves. CVJ is also approximated by critical vasculature supplying the brain. Congenital, developmental, and acquired disorders can affect CVJ, and surgical treatment remains challenging because of the complex anatomic and biomechanical characteristics of the region [102]. For many years, the microsurgical transoral technique has been applied as the standard method for the anterior approach to CVJ [103]. However, bacterial contamination, postoperative nasogastric tube feeding, and swelling of the tongue have been reported with this approach [104,105]. Recently, several endoscopic methods were reported regarding approaches to CVJ due to the advancement of endoscopic techniques [106,107] (Table 5 and Figure 7).

### 6.1. Endoscopic Endonasal Approach (EEA)

The endoscopic endonasal approach (EEA) to CVJ was first reported by Kassam et al. in 2005 [106]. The advantages of this approach are minimal invasiveness, unlimited surgical access to the cranial midline CVJ, and avoidance of palatal split. EEA is superior when the CVJ lesion exceeds the upper limit of the inferior third of the clivus [107]. EEA is also suited for ventral skull base lesions because it enables clear visualization of anterior neurovascular structures and reduces overall surgical morbidity [108]. In a comparative study of sinonasal malignancies, EEA has acceptable morbidity with low complication rates and can provide an oncologically sound alternative to open approaches [109].

### 6.2. Endoscopic Transoral Approach (ETA)

The first endoscopic transoral approach (ETA) was reported in 2004 [110]. Yadav et al. reported excellent results of 34 patients who underwent ETA [111]. The use of an endoscope with image guidance offered several advantages to provide access to the lower clivus and the C1-C2 region. This approach is becoming an emerging option to standard microsurgical techniques for transoral approaches to CVJ because of the wider working channel than EEA. Furthermore, ETA gains more consensus because the literature focuses more on EEA-related side effects [112].

### 6.3. Endoscopic Transcervical Approach

The endoscopic transcervical approach has the advantage of reducing the risk of cerebrospinal fluid leakage, maintaining a sterile surgical field, and providing an excellent surgical field for lower than C2. However, the indication of this approach is relatively limited because the cadaveric study proved that the endoscopic exposure of the high anterior cervical area was very difficult [113]. The endoscopic transcervical approach is not so popular because there are potential complications, like injuries to cranial VII, IX, XII, superior laryngeal nerve, the carotid artery, and cervical instability [112].

## 7. Latest Advances

### 7.1. Navigation

**Figure 8:**
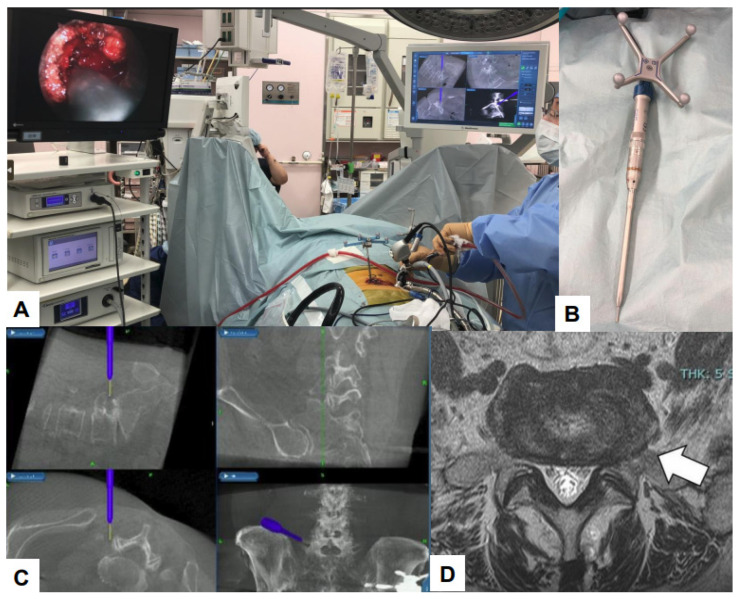
C arm free MED posterolateral approach. (**A**) Intra-operative image, (**B**) Navigated high-speed burr, (**C**) Navigation monitor, (**D**) Preoperative MRI, A white arrow shows left L5/S1 lateral lumbar disc herniation.

**Figure 9:**
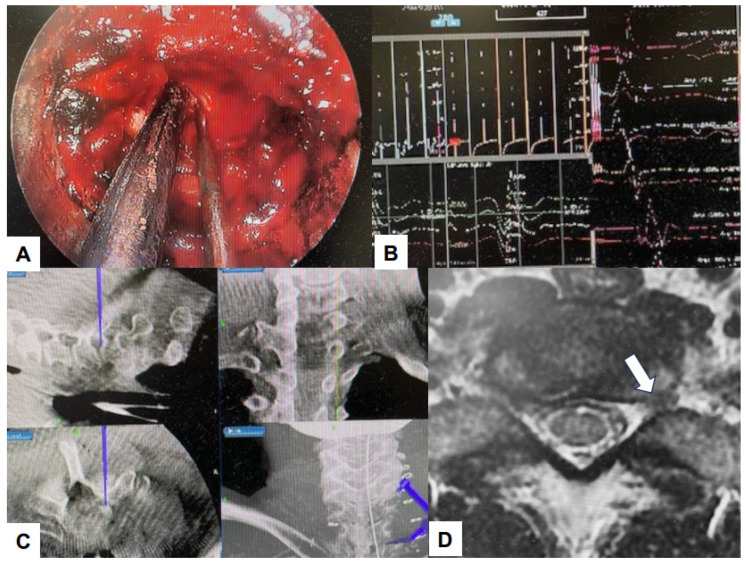
C arm free cervical endoscopic keyhole foraminotomy. (**A**) Endoscopy image, (**B**) Neuromonitoring, (**C**) Navigation monitor, (**D**) MRI. A white arrow shows cervical disc herniation.

**Figure 10:**
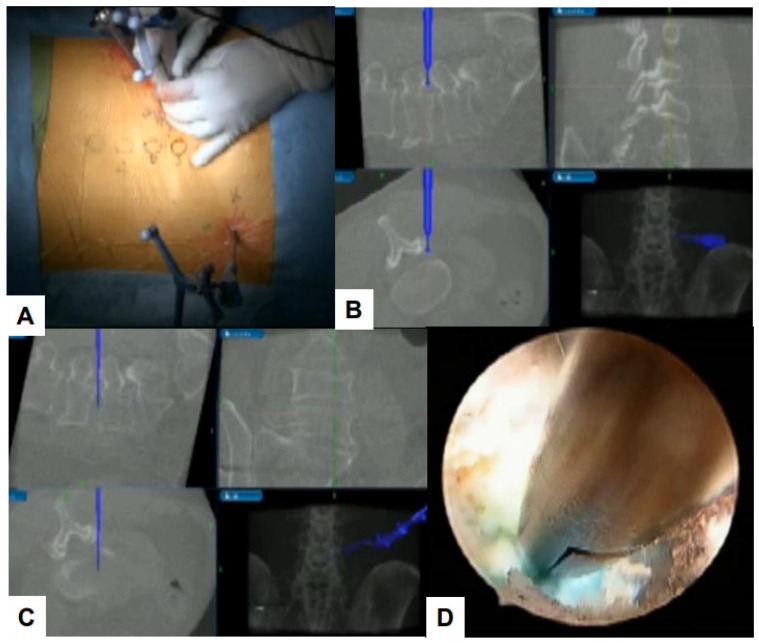
C arm free lumbar transforaminal discectomy. (**A**) Endoscopy image, (**B**) Foraminoplasty with a navigated high-speed burr, (**C**) Navigated approach to the disc, (**D**) Endoscopy image.

**Figure 11:**
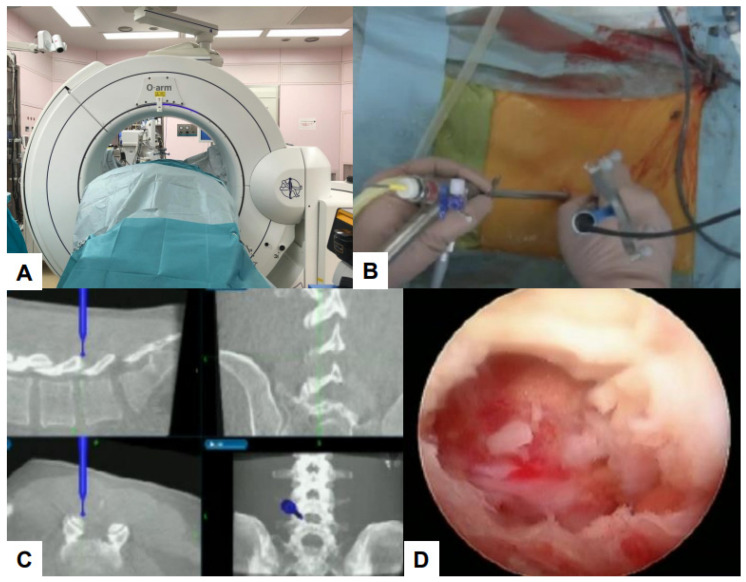
C arm free UBE. (**A**) O-arm, (**B**) Intra-operative image, (**C**) Navigation monitor, (**D**) endoscopy image.

Debilitating consequences for patients can occur due to damage to important structures such as nerves and blood vessels. Navigation systems that use real-time images improve surgical accuracy [114,115]. Using O-arm, a high-quality computed tomography (CT) scan can be performed. Surgeons can perform intra-operative three-dimensional (3D) anatomical mapping in real time by integrating it with a real-time anatomical tracking tool [116]. Spine surgeons initially used navigation systems for percutaneous pedicle screw fixation, but presently, navigation systems can be used for various surgical procedures [117]. Some examples of such applications in the field of endoscopic spine surgery are shown in the figures below (Figure 8, Figure 9, Figure 10 and Figure 11).

### 7.2. Ultra-Resolution and Three-Dimensional Endoscopes

Resolution refers to the number of pixels a display holds. For instance, 2K (Full HD) has a resolution of 1920 × 1080 pixels, whereas 4K (Ultra HD) has a resolution of 3840 × 2160 pixels. The advantage of higher resolutions is their capacity to depict patient tissues with greater precision [118]. Also, the current 2-dimensional endoscopic spine systems lack depth perception, causing unfamiliarity with surgical anatomy and may cause devastating complications [119]. Three-dimensional (3D) endoscopic equipment provides clear views of surgical anatomy, such as exposure of dura and nerve roots [120]. Using a 4K ultra-resolution endoscope, structures such as foraminal ligaments, which are difficult to observe with conventional microscopes, can be easily identified [121]. Furthermore, accurate perception of the degree of stenosis and disc protrusion using 3D visualization could reduce surgical uncertainty, followed by better decompression of neural structures and better surgical outcomes [119].

### 7.3. Robot-Assisted Endoscopic Surgery

Robot-assisted spine surgery with systems such as Mazor X (Medtronic Inc., Dublin, Ireland) and ROSA (Medtech S.A., Montpellier, France) is being performed especially for pedicle screw placement with several advantages [122,123]. This technology enables surgeons to enhance their manual dexterity with greater control and maneuverability through even a small portal, reducing physiological tremors [124]. Robot-assisted spine surgery can provide accurate and safe guidance for discography in the initial steps of percutaneous endoscopic cervical discectomy or PELD [125,126]. Robot assistance was recently used to perform full endoscopic lumbar discectomy [127]. Thus, robot-assisted surgery may be used to perform other techniques of spine endoscopy in the near future.

## 8. Conclusions

The field of endoscopic spine surgery has been developing rapidly over the last 40 years. Though initially limited to lumbar discectomies, endoscopic spine surgeries have been used to treat cervical and thoracic stenosis as well. Based on the current research, it is evident that minimally invasive techniques, such as endoscopic spine surgery, have fewer complications compared to conventional spine surgery. Thus, these techniques can be considered to be a safe and viable alternative to conventional spine surgery. However, the data regarding the applications to spinal conditions other than degenerative pathologies, such as spinal tumors, infection, and trauma, is lacking.

## Figures and Tables

**Figure 5:**
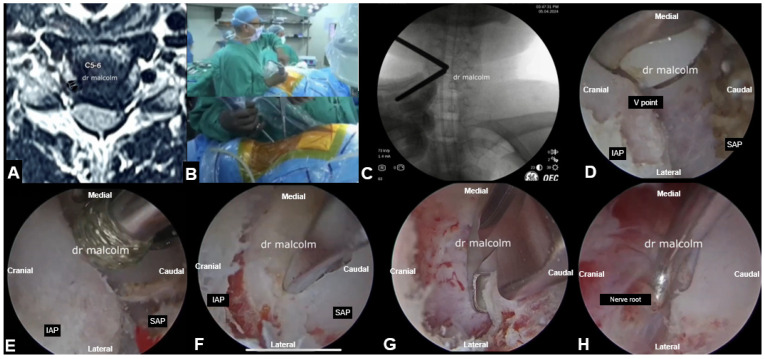
UBE cervical foraminotomy and laminectomy. (**A**)Axial section through C5-C6 disc showing left-sided disc osteophyte complex with impingement of left C6 nerve root, (**B**) Intra-operative surgical view, (**C**) Intra-operative fluoroscopic view showing targeting of V point, i.e., junction of inferior articular process (IAP) with superior articular process (SAP), (**D**) Endoscopic view showing identification of V point. (**E**) Beginning of decompression by burring of IAP (**F**,**G**) Resection of IAP and SAP with Kerrison rongeurs, (**H**) Final image showing adequate decompression of the nerve root.

**Figure 6:**
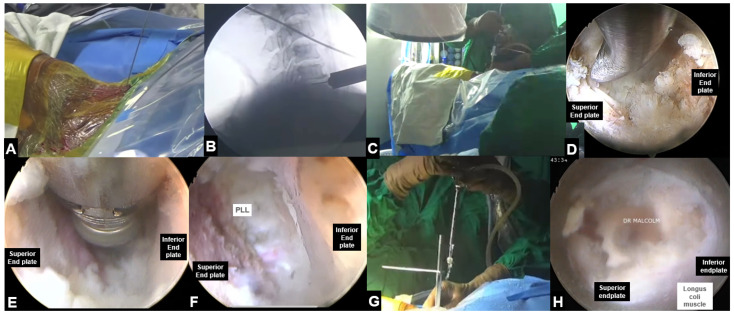
Endoscopic anterior cervical discectomy and fusion, (**A**) Intra-operative level marking, (**B**,**C**) Docking over C6-C7 disc space, (**D**,**E**) Endoscopic view showing removal of disc and preparation of disc space, (**F**) Endoscopic view showing complete removal of the disc and adequate endplate preparation, (**G**) Introduction of the cage, (**H**) final position of the cage.

**Table 3:** Clinical Results of Unilateral Biportal Endoscopy.

Author	Study Type	Sample	Diagnosis	Follow-Up (Months)	Operation Time (Minutes)	Complications
Eum et al. (2016) [27]	Retrospective	58	LSS	13.8	68.9 ± 16.1	13.8%; post-op headache (3), dural tear (2), transient numbness (2), epidural hepatoma (1)
Choi et al. (2016) [62]	Retrospective	68	LDH (25), Revision (3) stenosis (39), synovial cyst (1)	NR	68.2 ± 23.7	10.3%; dural tear (2), nerve root injury (1), incomplete decompression (4)
Kim et al. (2018) [63]	Retrospective	60	LDH	12.6	70.15 ± 22.0	5%, incomplete decompression (3)
Ahn et al. (2018) [55]	Retrospective	21	Foraminal stenosis (11), foraminal LDH (9), ASD (1)	14.8	96.7	4.8%; dural tear (1)
Kim and Choi (2018) [64]	Retrospective	105	LSS	14	53 ± 13.5	2.9%; dural tear (2), epidural hematoma (1)
Akbary et al. (2018) [65]	Retrospective	30	Lateral recess + foraminal stenosis	5.67	102.5 ± 43.66	0%
Pao et al. (2019) [66]	Retrospective	81	LSS	8.6	NR	8.6%; dural tear (4), transient motor weakness (1), inadequate decompression (1), epidural hematoma (1)
Wang et al. (2023) [67]	Prospective	70	LDH	24	NR	0%

**Table 4:** Clinical Result of Cervical Spine Endoscopy.

Author	Sample	Approach	Follow-Up	Outcomes	Complications
Fontanella (1999) [96]	296 (273 anterior,23 posterior)	Anterior, posterior	12 months	97% success at 1 year; average surgical time 25 min	Nil
Ahn (2005) [97]	111	Anterior	49 months	Excellent/good outcome in 80%	1 persistent radicular pain 1 patient required ACDF
Lee (2007) [98]	116	Anterior	36 months	87% had good outcomes	2 patients ACDF 2 patients repeat endoscopy 1 patient—posterior fixation
Reutten (2009) [99]	60	Anterior	24 months	96%—good clinical outcome	2 patients—progressive neck pain 2 patients—radicular pain 4 patients—ACDF
Yang (2014) [100]	84	Anterior 42Posterior 42	18 months	Shorter operating time for anterior approach Shorter hospital stay for posterior approach	1 neurological deterioration 1 hematoma 1 re-operation 1 postoperative headache
Oertel (2016) [101]	43	Posterior	6 months	82% regained full arm strength; mean one-level operation time 77 min	1 hematoma, 1 triceps paresis

**Table 5:** Three different endoscopic approaches to the craniovertebral junction.

	Endonasal	Transoral	Transcervical
Target	Livus, C1, upper C2	Clivus, C1, C2	Lower C2
Advantages	A better view, shorter surgical trajectory, wider working area, no tongue retraction	More familial anatomy, wide working area, no splitting of the soft palate	Reduced risk of CSF leakage, the sterile surgical field, fewer retraction complications
Disadvantages	A contaminated field, CSF leak management, a more limited caudal exposure	A contaminated field, a deeper surgical corridor, the airway swelling	Narrow angles, pharyngeal retraction, limitation by the chest on the angle of attack

## Data Availability

The data presented in this study are available in the article.
